# The Influence of Hearing Impairment on Mental Age in Down Syndrome: Preliminary Results

**DOI:** 10.3389/fped.2021.752259

**Published:** 2021-10-20

**Authors:** Amanda Saksida, Davide Brotto, Giulia Pizzamiglio, Elena Bianco, Sara Bressan, Agnese Feresin, Maura Bin, Eva Orzan

**Affiliations:** ^1^Institute for Maternal and Child Health - IRCCS “Burlo Garofolo”, Trieste, Italy; ^2^Associazione Sindrome di Down Onlus (AGBD), Centro di riabilitazione funzionale per disabili Verona, Verona, Italy; ^3^School of Medicine and Surgery, University of Verona, Verona, Italy

**Keywords:** down syndrome, mental age, hearing impairment (HI), auditory ability, receptive language ability

## Abstract

The increased life expectancy for patients with Down Syndrome (DS) has elicited the need to improve their quality of life by enhancing functional outcomes and identifying the factors that contribute to their long-term cognitive decline. Although the majority of individuals with DS have issues with hearing impairment (HI) since early childhood, to our knowledge no study has investigated whether HI represents a potential modulator of cognitive decline over time. The present explorative cohort study, albeit very preliminary due to the limited cohort (17 children), highlights the significant relation of a significant HI not only with receptive language abilities, but also with mental age in young patients with DS. Additional studies are required to confirm the link between HI and mental age and to assess the impact of audiological treatment on the enhancement of functional outcomes and of cognitive decline in individuals with DS.

## Introduction

The increased life expectancy for patients with Down Syndrome (DS) has elicited renewed interest in the factors that may contribute to their characteristic poor linguistic development, overall intellectual disability, and the developmental trajectory associated with long-term cognitive decline in this population (1, 2). In adult population with DS, sleep quality, changes in emotional/behavioral functioning, gait dyspraxia, and language skills havechanges in emotional/behavioral functioning, gait dyspraxia, and language skills have been identified as important predictors of cognitive decline (2–6). In parallel, various factors have been observed to affect cognitive function in pediatric population with DS, such as obstructive sleep apnea (OSA) and the history of cardiac interventions (HCI) (7–9). Whereas we know too little about the overall developmental trajectories of cognitive decline in DS, it has been recently shown that intelligence, adaptive functioning, and motor skills in adolescents with DS are strongly correlated with their mental age in early childhood and that congenital heart defects may have a negative impact on language outcomes (10, 11). Therefore, understanding cognitive functioning early in life may be important in charting cognitive decline over time (1, 12).

A possible detrimental factor for language abilities is hearing impairment (HI) (13), present in up to 90% of patients with DS (14). It is unknown, however, whether the HI may have a direct impact also on the cognitive performance and on mental age growth in these patients. The aim of the present study is to investigate the attested influence of HI on auditory and receptive language skills in young children with DS, prior to their audiological care/rehabilitation. Furthermore, the influence of HI on mental age in the sample was explored, as well as other two factors measured in the sample that are known to affect cognitive abilities in pediatric population with DS, obstructive sleep apnea (OSA) (7) and the history of cardiac interventions (HCI) (8).

## Materials and Methods

### Participants

Among 150 children and adolescents with DS who were involved in the rehabilitation process at the “*Associazione Sindrome di Down Onlus*” rehabilitation center in Verona (Italy) at the end of 2019, 41 were referred for audiological evaluation at the Audiology Unit of the *Institute for Maternal and Child Health IRCCS Burlo Garofolo* of Trieste (Italy) because, for various medical or family reasons, they had not had previous access to audiological treatment. In order to obtain a more homogeneous group of young but nonetheless collaborative subjects who underwent auditory and language tests, we excluded children younger than 3 and older than 10 years, with native language other than Italian, with serious vision problems, and with severe cognitive (IQ < 40) or communication deficits (i.e., autism spectrum disorder). The final dataset thus included 17 patients (nine girls, eight boys, mean age = 5.84 years; SD = 2.12, range: 3–10 years).

Parents were informed about the possibility to use clinical data for research purposes and gave their written consent to participate before the assessment. The study was conducted according to the 1964 WMA Helsinki declaration and its later amendments.

### Testing Procedure

All evaluations and data were obtained by a team of clinicians and therapists with specific expertise in DS and with particular attention to the audibility of the test stimuli. In addition to the audiological evaluation, auditory and receptive language skills, and cognitive abilities were measured. The information on chronological age, mental age, IQ, OSA, and HCI were collected from medical reports.

Audiometry testing was performed in a soundproof booth; sound field air conduction hearing threshold was estimated and averaged over frequencies of 0.5, 1, 2, and 4 kHz. Participants with either sensorineural or conductive hearing loss and with a mean hearing threshold >40 dB HL were categorized as having a significant HI (SHI). Auditory skills in everyday life were categorized using the Categories of Auditory Performance (CAP), an index consisting of eight scores arranged in order of increasing difficulty (15). Receptive language skills were assessed by administering the relative subtest of the Battery for the assessment of language in children aged 4 to 12 (BVL_4-12) (16). Percentage of correct responses was reported for each child. Nonverbal mental age and cognitive function (IQ) were estimated with the Leiter-R (17), an individually administered test designed to assess nonverbal cognitive functions in ages 2–20. Mental age was estimated from the W-scale estimate of ability and chronological age from a W-scale (a Rasch-based score) age equivalence for each month of age (18). OSA-related neurobehavioral morbidity was assessed with the 22-item Sleep Related Breathing Disorder scale of the Pediatric Sleep Questionnaire (PSQ) (7). A score higher than 8 was associated with the presence of pediatric OSA (7).

### Data Analysis and Availability

To describe the sample, IQ, PSQ scores, mental and chronological age, auditory performance and receptive language scores were compared in children with or without SHI using a series of Welch two sample *t*-tests controlled for multiple comparisons. Welch *t*-tests were used to control for the unequal variances of the sample.

To assess possible relations between mental age and factors that potentially affect cognitive function, bivatiate correlations (Pearson method) between mental and chronological age in children with and without SHI were computed, as well as the correlations in children with and without HCI and OSA. Partial correlations were not possible since splitting variables were categorical, whereas multiple regression analysis did not result in constructing valid models due to the scarcity of the data. The anonymised dataset is publicly available at https://osf.io/chw8n/.

## Results

Nine out of 17 children in the dataset were affected by a SHI. Children with and without SHI did not differ in chronological age, mental age and IQ, the factors that otherwise affect auditory and language skills (Table 1). Conversely, children with SHI had significantly lower results in auditory performance (CAP) and marginally significantly lower results in receptive language scores (BVL) (Table 1). BVL scores, however, showed significant positive correlation with the mental age in children without SHI [R = 0.7, t (6) = 2.399, *p* = 0.05], but not in children with SHI [R = 0.422, t (7) = 1.233, *p* = 0.258]. These results confirm that the presence of SHI may crucially affect auditory and language abilities in children with DS. Chronological age, mental age, IQ, CAP, and BVL scores were furthermore compared when children were split based on the presence or absence of OSA or HCI, but the differences were not significant.

**Table 1:** Mean mental age, chronological age, IQ, Pediatric Sleep Questionnaire (PSQ) scoring, auditory performance category (CAP), and receptive language (BVL) scoring in patients with and without an SHI, and the results of the non-parametric *t*-tests of the differences between the two subgroups.

**HI**	**Patients without SHI (*N* = 8)**	**95% CI**	**Patients with SHI (*N* = 9)**	**95% CI**	**t**	**df**	***p*-value**	**Eff. size (Cohen's d)**
Mean hearing threshold (dB HL)	27.5	24.15–30.85	46.111	38.91–53.31	−5.429	11.1	<0.001	2,52
IQ	64.125	51.95–76.30	54.667	46.79–62.54	1.531	12.412	0.151	0,76
mental age (months)	39.125	33.66–44.59	34.889	30.04–39.74	1.356	14.626	0.196	0,66
chronological age (months)	67.875	47.49–88.26	72	50.72–93.28	−0.327	15	0.748	0,16
PSQ score	7	2.98–11.02	9.3	5.85–12.81	−1.026	14.498	0.322	0,50
CAP	5.375	3.59–7.16	2.667	1.73–3.61	3.157	10.883	0.009	1,58
BVL	58.25	35.64–80.86	36.222	23.85–48.59	2.009	11.133	0.069	1,01

**Figure 1:**
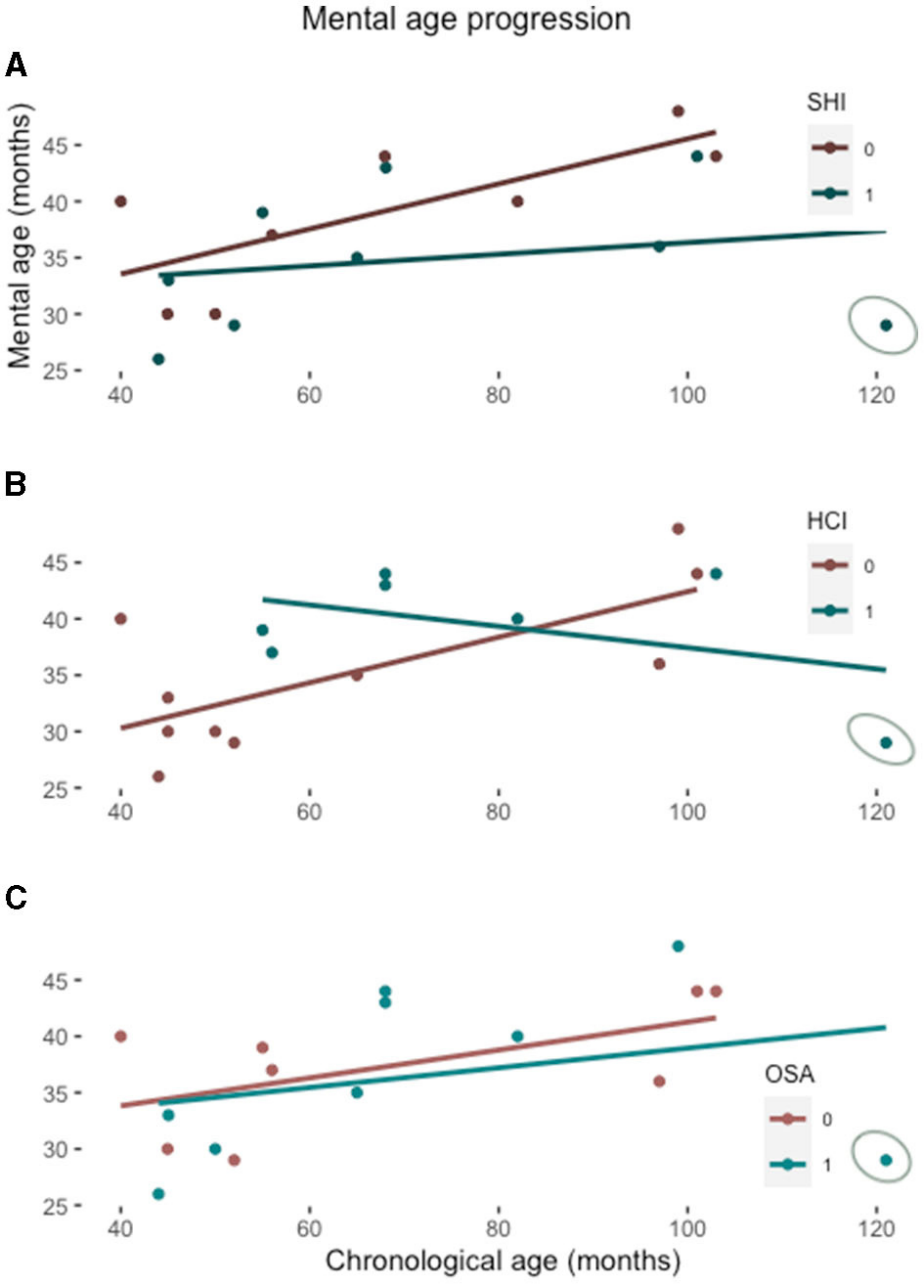
Scatterplot showing the mental age relative to the chronological age in patients with and without a SHI **(A)**, with and without the history of cardiac interventions (HCI) **(B)**, and with and without OSA **(C)**. The lines represent the linear regression function for each subgroup. The patient with all three conditions overlapping is circled on each graph.

To assess the potential role of SHI to the mental age growth, we have run separate correlation analyses between mental and chronological age for children with and without SHI. In children without SHI, mental age is positively correlated with the chronological age [R = 0.75, t (6) = 2.752, *p* = 0.033], indicating that mental age progresses steadily with growing. Conversely, children with SHI exhibited no significant correlation between the mental and chronological age [R = 0.227, t (7) = 2.752, *p* = 0.556], indicating the lack of progress in intellectual performance relative to the chronological age (Figure 1A). Mental age is also positively correlated with the presence of HCI[R = 0.723, t (8) = 2.961, *p* = 0.018], but not with the presence of OSA (Figures 1B,C). However, there is little overlap between SHI and HCI or OSA; in fact, five of nine children with SHI have been identified with OSA and there is no difference between the PSQ scores in children with and without SHI (Table 1); furthermore, only three of nine children with SHI have had HCI. We therefore conclude that their relation to the mental age may be independent.

## Discussion

The present study highlights the significant impact of SHI not only on auditory and receptive language abilities, but also on mental age growth in young patients with DS.

Our results confirm data from the previous DS cohort report in which children with DS and a history of HI had lower receptive and productive language skills than children without such a history (13). They also partially confirm previous reports on the impact of OSA and heart diseases on neurocognitive development of children with DS (7, 8). Possibly, co-morbidity of these factors leads to further decline in neurocognitive functions, as indicated by the case with all three conditions (Figure 1). The novel contribution of the present study, however, is the result indicating that the growth trend of mental age in children with DS may be reduced also by the effect of SHI. These, albeit very preliminary data could add a new perspective on the management of intellectual disability in DS, emphasizing the role of early diagnosis and treatment of HI in order to minimize the factors that may have a detrimental impact on mental and intellectual abilities in DS later in life. This is particularly relevant considering the high prevalence of HI (14) and the progeroid course in DS characterized by early dementia (19).

Growing literature emphasizes the impact of HI on the outset and progression of dementia, but very little is known about its role in DS. The impact of SHI to mental age growth at an early age could have long-lasting consequences on autonomy and quality of life, and possibly also on the outset and progression of dementia in DS. Due to the numerically very limited cohort, the current results should be considered preliminary. Further research is needed to clarify whether hearing deprivation due to SHI represents a causal factor or a mediator of cognitive functioning in individuals with DS. Future longitudinal studies could concurrently evaluate the impact of audiological treatment on improving functional outcomes and progression of cognitive decline in individuals with DS (1).

HI is a modifiable condition, and effective audiological treatment and rehabilitation are today feasible since the first months of life, minimizing the hearing deficit and its consequences. Yet, audiological assessment of patients with DS is frequently complex and severely delayed as a consequence of multiple life-threatening conditions that influence the clinical management in the first years of life. Nonetheless, professionals involved in the healthcare management of these patients should consider the possible presence of HI as a red-flag not only of a language disorder but possibly also of reduced mental age growth.

## Data Availability Statement

The datasets presented in this study can be found in the OSF repository at the following address: https://osf.io/chw8n/.

## Ethics Statement

Ethical review and approval was not required for the study on human participants in accordance with the local legislation and institutional requirements. Written informed consent to participate in this study was provided by the participants' legal guardian/next of kin.

## Author Contributions

AS, DB, GP, and EO: study concept and design. EB, SB, AS, DB, and GP: acquisition, analysis, and interpretation of data. DB, AF, MB, and GP: drafting of the manuscript. EO: study supervision. All authors had full access to all the data in the study and take responsibility for the integrity of the data and the accuracy of the data analysis.

## Conflict of Interest

The authors declare that the research was conducted in the absence of any commercial or financial relationships that could be construed as a potential conflict of interest.

## Publisher's Note

All claims expressed in this article are solely those of the authors and do not necessarily represent those of their affiliated organizations, or those of the publisher, the editors and the reviewers. Any product that may be evaluated in this article, or claim that may be made by its manufacturer, is not guaranteed or endorsed by the publisher.
